# Proteolytic Activities of Enterovirus 2A Do Not Depend on Its Interaction with SETD3

**DOI:** 10.3390/v14071360

**Published:** 2022-06-22

**Authors:** Xiaoyao Yang, Chiara Aloise, Arno L. W. van Vliet, Marleen Zwaagstra, Heyrhyoung Lyoo, Anchun Cheng, Frank J. M. van Kuppeveld

**Affiliations:** 1 Virology Section, Infectious Diseases and Immunology Division, Department of Biomolecular Health Sciences, Faculty of Veterinary Medicine, Utrecht University, 3584 CL Utrecht, The Netherlands; x_yang001@163.com (X.Y.); c.aloise@uu.nl (C.A.); a.l.w.vanvliet@uu.nl (A.L.W.v.V.); m.zwaagstra@uu.nl (M.Z.); h.r.lyoo@uu.nl (H.L.); 2 Institute of Preventive Veterinary Medicine, College of Veterinary Medicine, Sichuan Agricultural University, Chengdu 611130, China

**Keywords:** SETD3, CVB3 2A^pro^, nucleocytoplasmic traffic disorder, stress granules, interferon

## Abstract

Enterovirus 2A^pro^ is a protease that proteolytically processes the viral polyprotein and cleaves several host proteins to antagonize host responses during enteroviral infection. Recently, the host protein actin histidine methyltransferase SET domain containing 3 (SETD3) was identified to interact with 2A^pro^ and to be essential for virus replication. The role of SETD3 and its interaction with 2A^pro^ remain unclear. In this study, we investigated the potential involvement of SETD3 in several functions of 2A^pro^. For this, we introduced the 2A^pro^ from coxsackievirus B3 (CVB3) in a mutant of encephalomyocarditis virus (EMCV) containing an inactivated Leader protein (EMCV-L^zn^) that is unable to shut down host mRNA translation, to trigger nucleocytoplasmic transport disorder (NCTD), and to suppress stress granule (SG) formation and type I interferon (IFN) induction. Both in wt HeLa cells and in HeLa SETD3 knockout (SETD3^KO^) cells, the virus containing active 2A^pro^ (EMCV-2A^pro^) efficiently cleaved eukaryotic translation initiation factor 4 gamma (eIF4G) to shut off host mRNA translation, cleaved nucleoporins to trigger NCTD, and actively suppressed SG formation and IFN gene transcription, arguing against a role of SETD3 in these 2A^pro^-mediated functions. Surprisingly, we observed that the catalytic activity of enteroviral 2A is not crucial for triggering NCTD, as a virus containing an inactive 2A^pro^ (EMCV-2A^m^) induced NCTD in both wt and SETD3^KO^ cells, albeit delayed, challenging the idea that the NCTD critically depends on nucleoporin cleavage by this protease. Taken together, our results do not support a role of SETD3 in the proteolytic activities of enterovirus 2A^pro^.

## 1. Introduction

The family *Picornaviridae* comprises a large group of small non-enveloped positive-sense single-stranded RNA viruses. The picornaviral genome is about 7–8.5 kb in length, which is composed of a single open reading frame and two untranslated regions (UTRs) at the 5′ and 3′ ends. During infection, the viral genome is rapidly translated into a single polyprotein harboring the P1 region capsid proteins and the P2 and P3 region non-structural proteins. This polyprotein is proteolytically processed into mature viral proteins by viral proteases [1].

The picornaviruses family include important pathogens, causing a wide range of disease in humans and animals. The genus *Enterovirus* is one of the many genera of this family. This genus can be further divided into 15 species, *Enterovirus A*-*L* and *Rhinovirus A*-*C*. Among these species, numerous serotypes have been identified [2]. Well-known enteroviruses are poliovirus, coxsackievirus, and echovirus. Enteroviruses produce three viral proteases 2A^pro^, 3CD^pro^, and 3C^pro^, which process the viral polyprotein into mature proteins. 2A protein is a chymotrypsin-like cysteine proteinase that is responsible for the cleavage at the P1/P2 junction, whereas 3C^pro^ and 3CD^pro^ are responsible for the other cleavage events in the viral polyprotein.

Except for polyprotein processing, enteroviral 2A^pro^ also has diverse functions in suppressing host antiviral responses. For instance, 2A^pro^ cleaves eIF4G to shut off translation of capped host cell mRNA [3,4], which happens at a very early time post infection [5], without impacting viral translation which is initiated by a cap-independent binding of ribosomes to the internal ribosome entry site (IRES) in the 5′UTR of the viral genome.

Nuclear pore complexes (NPC), which regulate the transport of host cargos between the nucleus and the cytoplasm, are other key targets of 2A^pro^ during infection [6]. 2A^pro^ cleaves nucleoporins (Nups), particularly Nup98 and Nup62, to disrupt the NPC [7,8]. Like eIF4G cleavage, Nup98 cleavage happens rapidly in infected cells, often within 2 h [5]. 2A^pro^ from different enteroviruses attack NPC at different rates and with different affinities [9,10]. The cleavage of Nups by 2A^pro^ induces a NCTD, disturbing the cargo shuttle and antiviral signaling between the nucleus and cytoplasm.

Host antiviral innate immunity is the first barrier against viruses. Enteroviruses have evolved many strategies to antagonize the innate immunity as well [11]. 2A^pro^ plays important roles in suppressing the formation of SGs and type I IFN induction. Several stress kinases have been identified including the protein kinase R (PKR), a well-known kinase that is activated in virus-infected cells by recognizing dsRNA [12,13,14,15]. Activation of PKR leads to the phosphorylation of eIF2α, which leads to interruption of cellular translation and the accumulation of SGs containing mRNAs that cannot be translated. Thus, these SGs contain stalled messenger ribonucleoprotein complexes, which can be translated again when the stress has been resolved. These SGs are also considered to function as a platform for antiviral signaling by RIG-I-like receptors such as RIG-I and MDA5 and downstream signaling molecules such as MAVS [16]. Enteroviruses have a profound effect on cellular stress signaling. In the early phase of enteroviral infection, 2A^pro^—by cleaving eIF4G and inhibiting cap-dependent host mRNA translation—triggers the formation of atypical SGs (aSGs), which contain some but not all SG marker proteins. Formation of these aSGs does thus not rely on PKR but is triggered by the shut-off of host translation by 2A^pro^. Later in infection, these aSGs disappear. Strikingly, 2A^pro^—through its proteolytic activity—is also responsible for preventing the formation of typical SGs, through a mechanism that is poorly understood but that may involve the disruption of the eIF4GI–G3BP interaction [17,18]. Consequently, in a virus with a proteolytically inactive 2A^pro^, no aSGs are formed but infection leads to the formation of tSGs, which are triggered by PKR-mediated eIF2α phosphorylation [17,18]. Thus, 2A^pro^ plays an important role in the regulation of SGs (i.e., induction of aSGs and suppression of tSGs). In addition, 2A^pro^ can directly cleave MDA5 and MAVS resulting in an efficient blockade upstream of IFN gene transcription [19,20]. By inhibiting translation of receptors and downstream signaling molecules, 2A^pro^ also attenuates type I IFN and type III IFN signaling [21,22,23].

Recently, the histidine *N*-methyltransferase SET domain containing 3 (SETD3), which methylates actin [24,25], was identified to interact with the enteroviral 2A^pro^ [26]. This interaction, which is independent of either the methyltransferase activity of SETD3 or the protease activity of 2A^pro^, was shown to be critical for replication of a broad panel of enteroviruses [26]. Methylation of actin also does not seem to be important for enterovirus replication, as reconstitution of SETD3 knockout cells with a methyltransferase-activity deficient SETD3 mutant could completely restore EV replication [26]. Therefore, the role of SETD3 in enterovirus replication remains enigmatic.

**Figure 1:**
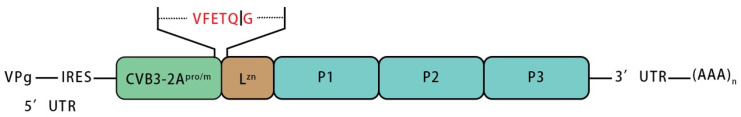
Schematic representation of EMCV-L^zn^ viral genome encoding CVB 2A^pro^ or inactivated CVB3 2A^m^. The EMCV leader protein (L) was inactivated by mutating its Zn-finger domain. The CVB3 2A^pro^ (wt or inactive mutant 2A^m^) coding sequence was introduced upstream of the inactivated L protein. A 3CD cleavage site (VFETQ/G) was introduced between 2A^pro^ and L to allow efficient release of 2A^pro^.

Investigating the possible role of the SETD3-2A^pro^ interaction for the various functions of 2A^pro^ is challenging as enteroviruses with proteolytically inactivated 2A^pro^ are non-viable and wildtype enteroviruses cannot replicate in SETD3 KO cells. Unlike enteroviruses, replication of the cardiovirus EMCV does not rely on SETD3. Like 2A^pro^, EMCV L plays important roles in triggering NCTD in infected cells as well as in suppressing SG formation and IFN induction [27], although the underlying mechanisms differ as L is not a protease [28]. We previously showed that heterologous expression of CVB3 2A^pro^ in an EMCV recombinant (EMCV-2A^pro^) (Figure 1) [18], could functionally substitute for an inactive L protein containing mutations in its zinc-finger domain (L^zn^) [29,30]. Using EMCV-2A^pro^, we here show that the functions of 2A^pro^ to cleave eIF4G, trigger NCTD, and suppress SG formation and IFN induction do not rely on SETD3. Notably, during our studies we observed that catalytically inactive 2A^pro^ could still induce NCTD, albeit it was delayed, indicating that the virus-induced NCTD does not critically rely on the proteolytic activity of 2A^pro^.

## 2. Materials and Methods

### 2.1. Cells and Viruses

H1 Hela and H1 SETD3^KO^ cell lines were kind gifts from Jan E. Carette’s group. All Hela and Hela-derived cells were cultured in Dulbecco’s modified Eagle’s medium (DMEM) supplemented with 10% fetal calf serums and a mixture of penicillin and streptomycin. Recombinant EMCV viruses were derived from the pM16.1 infectious clone [31]. In EMCV-L^zn^, the stress and IFN antagonist (i.e., the Leader protein) is inactivated by the insertion of point mutations in its Zn finger domain (C19A/C22A). The genes encoding CVB3 2A^pro^ or 2A^m^ (mutation C109A) were introduced at the 5′ end of the EMCV [18]. The recombinant EMCV viruses mentioned in this study were recovered by transfection of RNA transcripts into BHK21 cells. The viruses were passaged on BHK21 cells. Once total CPE, cells were subjected to three freeze–thaw cycles and debris was pelleted at 4000× *g* for 15 min. Subsequently, the viruses were concentrated by ultracentrifugation through 30% sucrose cushion at 250,000× *g* for 16 h in an SW32Ti rotor and stored at −80 °C [18].

### 2.2. Antibodies

The primary antibodies that were used for IFA staining are: Mouse anti-dsRNA (English & Scientific Consulting, Szirák, Hungary), Rabbit anti-dsRNA (Absolute antibody, Oxford, UK); Mouse anti-hnRNP K (Santa Cruz, California, USA); Goat anti-eIF3 (Santa Cruz, California, USA); Rabbit anti-G3BP2 (Bethyl Laboratories, Texas, USA). The corresponding secondary antibodies that were used for IFA staining are: Alexa Fluor 488-, Alexa Fluor 594-, and Alexa Fluor 647-conjugated secondary antibodies (Invitrogen, California, USA). The primary antibodies that were used for WB are: Rabbit anti-eIF4G (Bethyl Laboratories, Texas, USA); Mouse anti-tubulin (Sigma, Andalucia, Spain). The monoclonal rat anti-2A antibody was a kind gift from Malin Flodström-Tullberg. Respective IRdye680- or IRdye800-conjugated secondary antibodies (LiCOR, Lincoln, NE, USA) were used for detection.

### 2.3. Quantitative Real-Time PCR (qRT-PCR Analysis)

Cells were seeded in 24-well plates and the next day infected with the indicated viruses at an MOI of 10. At the indicated time points, the cells were lysed and total RNA from cells was isolated using a total RNA isolation kit (Macherey-Nagel, Düren, Germany) according to the manufacturer’s instructions. Reverse transcription was set up using TaqMan reverse transcription reagents (Applied Biosystems, California, USA) before performing qPCR analysis with SYBR green (Roche, Basel, Switzerland). Data shown are the relative abundance of the indicated mRNA normalized to actin. The primers for IFN detection are: *Fw_5′-ATGACCAACAAGTGTCTCCTCC-3′; Rv_*5*′-GCTCATGGAAAGAGCTGTAGTG-3′.* The primers for actin detection: *Fw_5′-CCTTCCTGGGCATGGAGTCCTG-3′; Rv_5′- GGAGCAATGATCTTGATCTTC-3′.*

### 2.4. Western Blot Analysis

The WB assay was performed as described previously [18]. Briefly, cells were seeded in 6-well plates and the next day infected with the EMCV viruses indicated above at MOI of 10. At the indicated time points, cells were released using trypsin, washed and cell lysates were prepared. 100 micrograms of protein from the cleared cell lysates were resolved using SDS-PAGE (Bio-Rad, Irvine, CA, USA), and the proteins were transferred to 0.2 μm-pore-size nitrocellulose membranes. The membranes were incubated overnight with primary antibody and for 30 min with the secondary antibody. The membranes were scanned using an Odyssey imager (LiCOR, Lincoln, NE, USA).

### 2.5. Immunofluorescence Analysis

IFA was performed as described previously [18]. Briefly, cells were grown on 12-mm glass coverslips and the next day infected with the viruses indicated above (MOI=10 or 50). At the indicated time points, cells were fixed in 4% paraformaldehyde for 30 min and then permeabilized using 0.1% Triton X-100. Samples were incubated with primary antibody for 1 h and incubated with secondary antibodies and DAPI for 30 min. Coverslips were mounted on microscopy slides with FluorSafe^TM^ reagent (Merck Millipore, Massachusetts, USA). Finally, cells were examined by confocal microscopy (Leica SPE-II, Wetzlar, Germany) and Leica Application Suite Advanced Fluorescence (LAS-AF, Wetzlar, Germany) software.

### 2.6. Quantification of Cells with SGs

After staining of the cells with the antibodies against proteins of interest, 250 cells were counted for quantifying the ratio of cells with SGs in each sample. GraphPad Prism 7 software was used for statistical analysis. Error bars represent standard deviations. One-way analysis of variance (ANOVA) analysis was used to calculate *p* values.

## 3. Results

### 3.1. Replication of EMCV-2A^pro^ in WT and SETD^KO^ Cells

**Figure 2:**
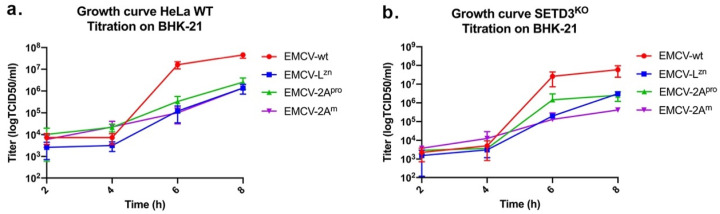
Recombinant EMCV-2A^pro^ replicates well in WT and SETD3^KO^ Hela cells. (**a**) Virus titers from WT Hela cells. (**b**) Virus titers from SETD3^KO^ Hela cells. Cells were infected with indicated viruses at an MOI of 5. Then cells were lysed at 2, 4, 6, and 8 hpi, respectively. Virus titers were determined by endpoint titration on BHK-21 cells. Three biological replicates were performed in each panel. One representative of two experiments is shown.

To verify that CVB3 is unable to infect SETD3^KO^ cells, we infected both WT Hela cells and SETD3^KO^ cells with CVB3-Rluc viruses at an MOI of 0.1. Then, *Renilla* luciferase activity was tested at 2, 4, 6, and 8 hpi. CVB3 infection was severely impaired in SETD3^KO^ cells (Figure S1), as shown previously [26]. Therefore, EMCV recombinant viruses carrying 2A^pro^ were used to study the importance of SETD3 for 2A^pro^ function. The construction and characterization of these viruses have been described previously [18]. Here, we set out to ensure that the recombinant EMCV-2A^pro^ viruses (wt 2A^pro^ and catalytically inactive mutant 2A^m^) could replicate well in the absence of SETD3. For this, single-cycle growth curves were determined in HeLa wt and SETD^KO^ cells. Both cell lines were infected with EMCV-wt, EMCV-L^zn^, EMCV-2A^pro^, and EMCV-2A^m^, a virus that contains inactivated 2A protein due to mutation C109A at an MOI of 5. Virus titers were determined at 2, 4, 6, and 8 h post infection (hpi), respectively, by endpoint titration (Figure 2). In both cell lines, EMCV-wt showed most efficient replication, whereas EMCV-L^zn^ showed delayed replication. EMCV-2A^pro^ also showed reduced replication but replicated slightly faster than EMCV-L^zn^ and EMCV-2A^m^. Similar virus replication kinetics were observed in WT and SETD3^KO^ cells. Therefore, these viruses represent a good model to investigate the function(s) of CVB3 2A^pro^ and the dependence on SETD3.

### 3.2. CVB3 2A^pro^ Expression and eIF4GI Cleavage in SETD3^KO^ Hela Cells

**Figure 3:**
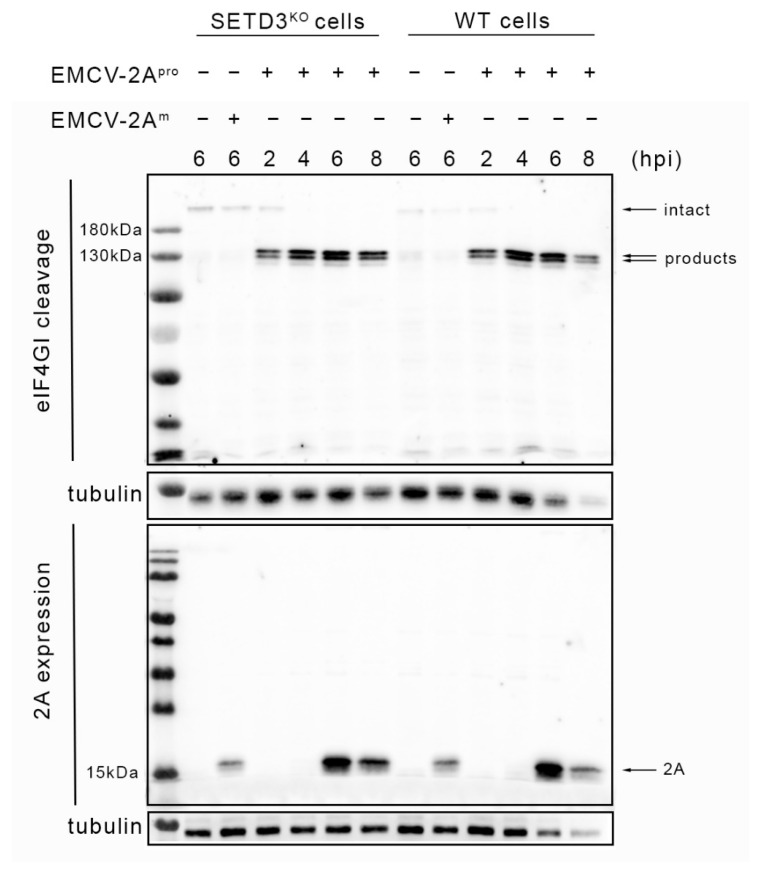
EIF4GI cleavage and 2A expression in WT and SETD3^KO^ cells. Cells were infected with EMCV-2A^pro^ viruses (MOI = 10). At 2, 4, 6, and 8 hpi, cells were collected and lysed in 100 μL lysis buffer. Proteins from both mock cells and cells infected with EMCV-2A^m^ were collected at 6 hpi. Cell lysates were subjected to SDS-PAGE and analyzed by Western blot analysis. Three independent experiments were performed.

Next, we tested whether the cleavage of eIF4GI by enteroviral 2A^pro^, which occurs early during infection, is dependent on SETD3. WT and SETD3^KO^ cells were infected with EMCV-2A^pro^ at an MOI of 10 and total cellular proteins were collected at 2, 4, 6, and 8 hpi. Additionally, cells infected with EMCV-2A^m^ at 6 hpi were included as a control. Western blot analysis was performed to detect eIF4GI cleavage and 2A expression (Figure 3). The molecular weight of intact eIF4GI is 220 kDa, and that of cleavage products are about 100 and 120 kDa, respectively [32]. In both cell lines, the infection of EMCV-2A^m^ did not result in the cleavage of eIF4GI (upper part). On the contrary, the cleavage of eIF4GI was evident already at 2 hpi in EMCV-2A^pro^-infected cells regardless of cell type. With time increasing, more eIF4GI cleavage products were observed, but little if any difference was observed between WT and SETD3^KO^ cells. The absence of SETD3 had no evident effect on the expression and stability of CVB3 2A^pro^ (lower part) as comparable amounts of 2A^pro^ were detected at 6 and 8 hpi in WT and SETD3^KO^ cells. Thus, SETD3 is not required for the CVB3 2A^pro^-mediated cleavage of eIF4GI during enteroviral infection.

### 3.3. SETD3 Is Not Required for the CVB3 2A^pro^-Triggered Nucleocytoplasmic Traffic Disorder

**Figure 4:**
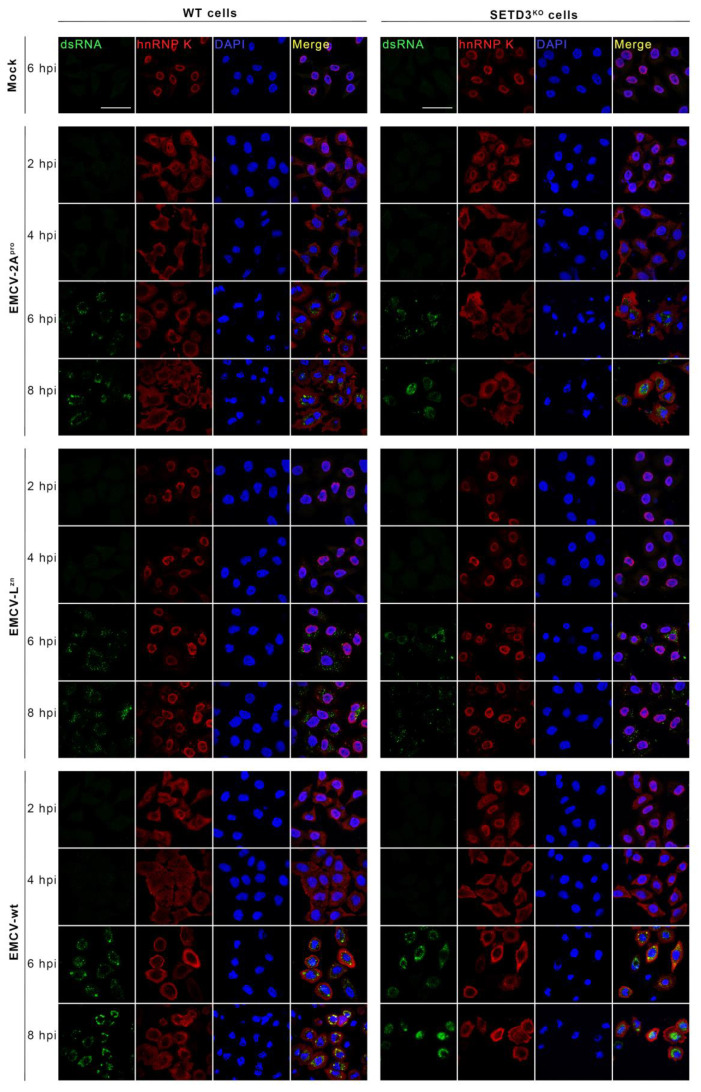
SETD3 protein is not required for NCTD induction by CVB3 2A^pro^. WT and SETD3^KO^ Hela cells were infected with indicated viruses at an MOI of 50. At 2, 4, 6, and 8 hpi, cells were fixed. Subsequently, IFA assays were performed with antibodies against hnRNP K, as a marker for NCTD, and against dsRNA, as a marker for viral RNA replication. The uninfected mock was collected from 6 hpi. Scale bar represents 10 μm, all images are in the same scale. Three independent experiments were performed.

Another important event, which happens early during enteroviral infection, is the 2A^pro^-mediated cleavage of nucleoporin proteins [5]. This leads to an NCTD that is characterized by the efflux of nuclear proteins and influx of cytosolic proteins into the nucleus. To investigate whether SETD3 is required for the CVB3 2A^pro^-induced NCTD, WT and SETD3^KO^ cells were infected with EMCV-2A^pro^, EMCV-L^zn^, and EMCV-wt at an MOI of 50 and the localization of hnRNP K was monitored by IFA at 2, 4, 6, and 8 hpi. hnRNP K is a scaffolding protein that constitutes the ribonucleoprotein (RNP) complexes of heterogeneous nuclear RNA in mammalian cells [33] and contains a classic nucleus localization signal (NLS) motif [34]. Additionally, an antibody against dsRNA was used as an indication of viral replication. EMCV-wt, which was included as a positive control, induced NCTD very early within 2 hpi in both cell lines, and from 6 hpi—a time point at which significant viral replication was detected—the hnRNP K proteins were mainly located in the cytosol (Figure 4). In cells infected with EMCV-L^zn^ virus, which was included as a negative control, the hnRNP K proteins always remained in the nucleus even in cells containing comparable amounts of dsRNA as observed in EMCV-wt-infected cells. Similar to EMCV-wt, EMCV-2A^pro^ rapidly induced NCTD within 2hpi. Efflux of hnRNP K protein was observed over time and the hnRNP K proteins localized almost exclusively in the cytoplasm from 6 hpi. No difference was detected between WT and SETD3^KO^ cells. Therefore, the SETD3 is also not important for CVB3 2A^pro^ to induce NCTD.

### 3.4. SETD3 Is Not Required for the CVB3 2A^pro^-Mediated Suppression of SG Formation

**Figure 5:**
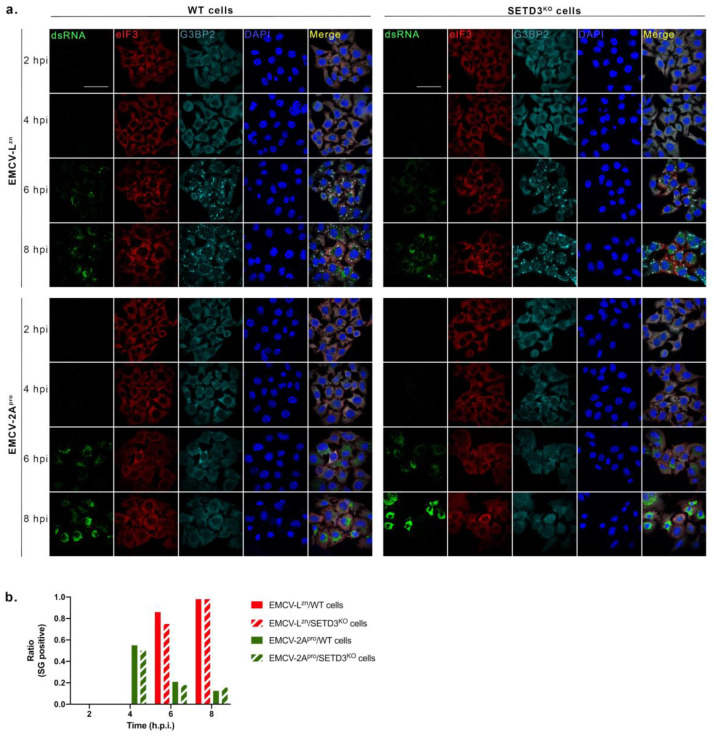
SETD3 protein is not required for the ability of CVB3 2A^pro^ to suppress SG formation. (**a**) WT and SETD3^KO^ Hela cells were infected with indicated viruses at an MOI of 10. At 2, 4, 6, and 8 hpi, cells were fixed. Subsequently, IFA assays were performed with antibodies against dsRNA, as a marker for viral RNA replication, and against eIF3 and G3BP2 as markers for SGs. Three independent experiments were performed. Scale bar represents 10 μm, all images are in the same scale. (**b**) Quantification of (**a**). In total, 250 cells were quantified in each sample. Graphs depict the ratio of SG-positive cells. The solid columns represent the ratio from WT cells and the diagonal columns represent the ratio from SETD3^KO^ cells.

Enterovirus 2A^pro^ suppresses SG formation through a mechanism that is poorly understood. Previously, we showed that infection of cells with EMCV-2A^pro^ leads to the appearance of atypical SGs (aSGs) early in infection due to cleavage of eIF4G and the ensuing inhibition of host cell mRNA translation. These aSGs disappear through a poorly understood function of 2A^pro^ and no typical SGs (tSGs) are observed in the mid and late phases of infection [18]. To investigate a possible involvement of SETD3 in this 2A^pro^ activity, we analyzed SG formation in WT and SETD3^KO^ cells infected with EMCV-2A^pro^. As a control, we included EMCV-L^zn^, which—unlike EMCV-2A^pro^—is unable to suppress SG formation and triggers the formation of typical SGs (tSGs) [18]. WT and SETD3^KO^ cells were infected with EMCV viruses at an MOI of 10, and SG formation was monitored at 2, 4, 6, and 8 hpi by IFA (Figure 5a). Antibodies against eucaryotic initiation factor 3 (eIF3) and RasGAP SH3-domain binding protein 2 (G3BP2) were used to detect SGs, and a dsRNA antibody was used to detect viral RNA replication.

The results show that EMCV-2A^pro^ infection triggered formation of aSGs at 4 hpi but no tSGs at later time points in either WT cells or SETD3^KO^ cells, whereas tSGs were observed in cells infected with EMCV-L^zn^, which is in line with the previous studies [18,35]. To accurately compare SG formation between WT and SETD3^KO^ cells, the occurrence of SGs was counted for 250 cells for each sample (Figure 5b). tSGs were formed in about 80% of cells at 6 hpi and almost all cells at 8 hpi during EMCV-L^zn^ infection, but EMCV-2A^pro^ effectively suppressed tSG formation in both WT and SETD3^KO^ cells. Therefore, the SETD3 protein is not required for the suppression of typical SGs by 2A^pro^.

### 3.5. CVB3 2A^pro^ Does Not Require SETD3 to Suppress Type I Interferon Induction

**Figure 6:**
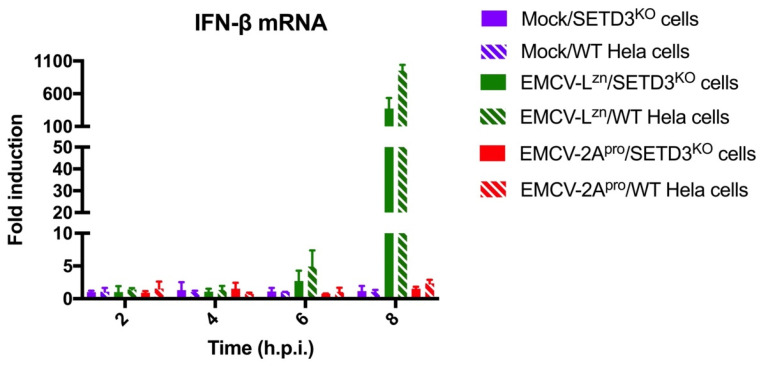
SETD3 protein is not required for the ability of CVB3 2A^pro^ to suppress IFN induction. WT and SETD3^KO^ Hela cells were infected with the indicated viruses at an MOI of 10. Cells were lysed at 2, 4, 6, and 8 hpi. Total RNA was extracted and used for RT-qPCR analysis for IFN-β and actin mRNA. The IFN-β levels are depicted as a fold induction compared to levels in uninfected mock cells, after correction for actin mRNA levels. The solid columns represent the levels from SETD3^KO^ cells, and the diagonal columns represent the levels from WT cells. Error bars depict the standard deviations. Three biological replicates were performed in each panel.

IFN induction is another key aspect of antiviral innate immune response [36]. Previously, we showed that enteroviruses strongly suppressed type I interferon (IFN) induction, most likely through the activity of 2A^pro^ to cleave MDA5 and MAVS [19]. Consistently, whereas EMCV-L^zn^ infection triggered a strong IFN response, this response was efficiently suppressed in cells infected with EMCV-2A^pro^ [18]. To investigate a possible role of SETD3 in the 2A^pro^-mediated suppression of IFN induction, WT and SETD3^KO^ cells were infected with EMCV-L^zn^ and EMCV-2A^pro^ at an MOI of 10. Total RNA was collected at 2, 4, 6, and 8 hpi and RT-qPCR analysis was performed to determine the IFN induction (Figure 6). The results showed that EMCV-L^zn^ infection induced significant IFN induction in both cell lines, but that EMCV-2A^pro^ did not trigger IFN induction in either WT or SETD3^KO^ cells. Therefore, SETD3 does not seem to be required by CVB3 2A^pro^ to suppress IFN induction.

### 3.6. CVB3 2A^m^ Protein Still Induces NCTD

**Figure 7:**
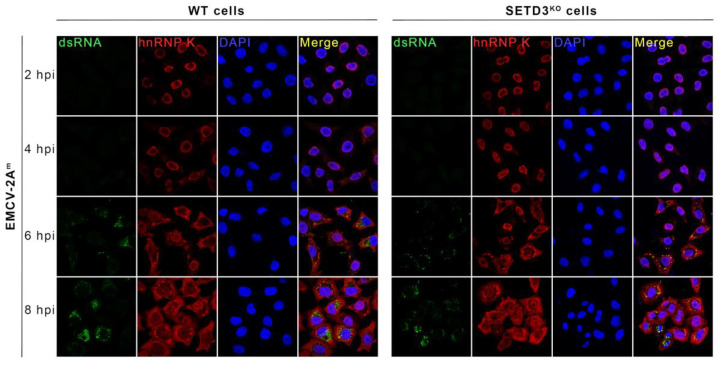
EMCV-2A^m^ infection induces NCTD in WT and SETD3^KO^ Hela cells. WT and SETD3^KO^ Hela cells were infected with EMCV-2A^m^ virus at an MOI of 50. At 2, 4, 6, and 8 hpi, cells were fixed. Subsequently, IFA assays were performed. Staining with hnRNP K antibody was performed to monitor NCTD and staining with dsRNA antibody was performed to monitor viral RNA replication. The uninfected mock was collected from 6hpi. Three independent experiments were performed.

We also tested the effect of EMCV-2A^m^ infection on NCTD in WT and SETD3^KO^ cells. We expected this proteolytically inactivate mutant not to induce NCTD, since NCTD was suggested to be highly dependent on the catalytic activity of 2A^pro^ [9,37]. Surprisingly, we found that CVB3 2A^m^ could still induce NCTD in both cell lines, albeit delayed (Figure 7). EMCV-2A^m^ infection induced hnRNP K protein efflux from 4 hpi onwards. At 8 hpi, hnRNP K protein efflux was detected in nearly all cells with no difference in WT and SETD3^KO^ cells. Consistent with our previous results (Figure 4), no hnRNP K efflux was observed in cells infected with EMCV-L^zn^ in this experiment (data not shown).

**Figure 8:**
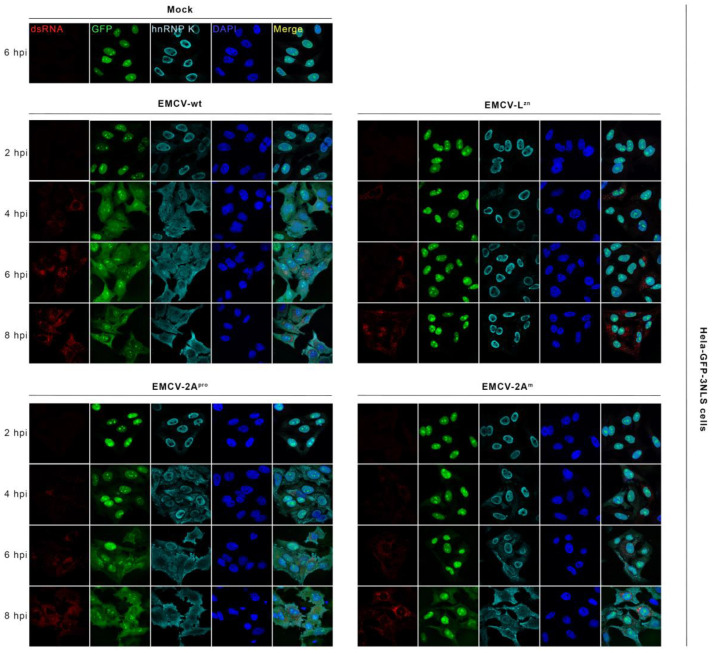
EMCV-2A^m^ infection induces NCTD in Hela-GFP-3NLS cells. Hela-GFP-3NLS cells were infected with indicated viruses at an MOI of 50. At 2, 4, 6, and 8 hpi, cells were fixed. Subsequently, IFA assays were performed with corresponding antibodies. The uninfected mock was collected from 6 hpi. DsRNA staining was performed as a viral replication marker. GFP fluorescence was observed and hnRNP K staining was performed as the NCTD marker. Three independent experiments were performed.

To further substantiate this unexpected finding, we performed similar experiments in Hela-GFP-3NLS cells stably expressing a GFP fused to three tandem nucleus localization signals (NLS). Hela-GFP-3NLS cells were infected with EMCV-wt, EMCV-L^zn^, EMCV-2A^pro^, and EMCV-2A^m^ at an MOI of 50 and the localization of both GFP-3NLS and hnRNP K was evaluated (Figure 8). All viruses replicated well in Hela-GFP-3NLS cells as indicated by similar levels of dsRNA. Upon infection of cells with EMCV-L^zn^, both GFP and hnRNP K protein remained in the nucleus, resembling mock-infected cells, whereas increasing efflux of hnRNP K and GFP-3NLS was observed starting from 2 hpi in cells infected with EMCV-2A^pro^. In cells infected with the EMCV-2A^m^, GFP-3NLS and hnRNP K located in the nucleus at an early time point, i.e., at 2 hpi, but from 4 hpi both proteins started to appear in the cytosol, in line with the results above in normal Hela cells.

Thus, for two different nuclear proteins, we showed for the first time that an enteroviral 2A catalytic-dead mutant was found to be able to induce NCTD, albeit with a delay. This finding challenges the previous notion that NCTD induced by enteroviral 2A protein is strictly dependent on its catalytic activity.

## 4. Discussion

Enterovirus 2A^pro^ is a protease that has several important functions in the viral life cycle. It cleaves at the P1-P2 junction of the polyprotein, it cleaves several host factors, such as eIF4G and nup proteins [5], and its proteolytic activity is important for suppressing type I IFN and antiviral stress responses [18,19,20]. Recently, SETD3 was identified as a novel essential host factor for enterovirus RNA replication. SETD3 was found to interact with 2A^pro^ [26] and mutations in 2A^pro^ that disrupt the interaction with SETD3 abolished viral RNA replication. However, the precise role of SETD3 during enterovirus replication remains unknown. SETD3 is an actin-specific histidine *N*-methyltransferase [24], but this activity is not required for its interaction with 2A^pro^ or virus replication. It is difficult to study the importance of SETD3-2A^pro^ interaction for any 2A^pro^ function as enteroviruses cannot replicate in SETD3 KO cells. Unlike enteroviruses, EMCV can replicate in SETD3 KO cells [26]. We previously generated an EMCV-2A^pro^ virus by inserting CVB3 2A^pro^ into the genome of a mutant virus in which the function of the L protein was disrupted, allowing us to demonstrate that CVB3 2A^pro^ suppresses SG formation and IFN induction through its proteolytic activity [18]. In this study, we infected SETD3 KO cells with this virus to gain insight into the importance of the SETD3-2A^pro^ interaction for the function(s) of 2A^pro^. Our results show that SETD3 is not required for the CVB3 2A^pro^-mediated cleavage of eIF4GI, the cleavage of nups and the ensuing NCTD, and the suppression of SG formation and IFN induction. Previously, we showed that the ability of 2A^pro^ to suppress SG formation and IFN induction is conserved among members of all human enterovirus species [18]. Therefore, we speculate that it is likely that SETD3 is dispensable for the function of other enteroviruses as well.

Having shown that SETD3 is not required for the known proteolytic functions of 2A^pro^, the importance of the SETD3-2A^pro^ interaction remains to be established. Previously, it has been shown that insertion of an IRES between P1 and P2 in the poliovirus genome yielded a viable recombinant virus [38]. This virus no longer relies on 2A^pro^ for cleaving at this junction, which is essential for virus replication. RNA transcripts containing mutation of C109A, an amino acid of the catalytic triad of 2A^pro^, failed to yield viable poliovirus, but these transcripts showed some RNA replication, albeit with reduced efficiency compared to RNA transcripts containing wt 2A^pro^. Recently, a viable EV-A71 recombinant virus was described containing an IRES between P1 and P2 and a catalytic inactive 2A^pro^, lending support that its proteolytic activity is dispensable for viral RNA replication [39]. In contrast, deletion of 2A^pro^ from the above-mentioned poliovirus RNAs containing an IRES between P1 and P2 rendered the RNA transcripts unable to replicate [38]. Together, these findings hint to the importance of a physical role of enterovirus 2A^pro^ in a yet unknown step in viral RNA replication, possibly involving an interaction with SETD3. The functional importance of the interaction of 2A^pro^ with SETD3 for viral RNA replication awaits further studies.

While performing our experiments, we made an unexpected observation with EMCV-2A^m^, a virus expressing a catalytic inactivated 2A^pro^. As expected, infection of cells with this virus did not result in eIF4G cleavage and this virus failed to suppress the IFN induction and SG formation. Strikingly, despite its inability to cleave host factors, infection with EMCV-2A^m^, but not with EMCV with an inactivated L protein lacking inactive 2A^pro^ (EMCV- L^zn^), triggered NCTD, albeit delayed. We cannot rule out that this unexpected finding is due to some functional interaction with an EMCV protein, although this possibility seems unlikely as we never observed any effect on NCTD by the EMCV-L^zn^ control virus. Together, these findings show that the proteolytic activity of 2A^pro^ is not absolutely required for NCTD induction. Notably, SETD3 is not important for 2A^pro^-triggered NCTD, as the same observations were made in SETD3^KO^ cells. These findings suggest that 2A^pro^ activity affects NCTD by two distinct mechanisms; one is through the cleavage of Nups, the other is through an unknown proteolytic activity-independent mechanism. Cardiovirus Leader proteins, which are devoid of any enzymatic activity, also trigger NCTD [40,41]. L protein binds directly to Ran, which regulates both nuclear import and export pathways [42]. This complex subsequently recruits activated kinase cargos leading to the hyperphosphorylation of Phe/Gly-containing Nups, thereby perturbing the integrity and functioning of NPCs and resulting in NCTD [28,43]. The Nup phosphorylation triggered by L protein is mediated by mitogen-activated protein kinases [44]. Recently, the Leader protein was shown to interact with mitogen-activated protein kinase p90-ribosomal S6-kinases (RSKs) and prevent their dephosphorylation, thereby maintaining the activity of kinases [45]. Whether 2A^pro^ also affects the activity of some kinases and thereby triggers Nup phosphorylation or, alternatively, whether it triggers NCTD through another mechanism requires further investigation.

## Data Availability

The data presented in this study are available in the insert article.

## Supplementary Materials

The following supporting information can be downloaded at: https://www.mdpi.com/article/10.3390/v14071360/s1, Figure S1: CVB3 infection in HeLa wt and SETD3^KO^ cells.

## References

[1] Yi E.J., Shin Y.J., Kim J.H., Kim T.G., Chang S.Y. (2017). Enterovirus 71 infection and vaccines. Clin. Exp. Vaccine Res..

[2] Filipe I.C., Guedes M.S., Zdobnov E.M., Tapparel C. (2021). Enterovirus D: A small but versatile species. Microorganisms.

[3] Lloyd R.E., Grubman M.J., Ehrenfeld E. (1988). Relationship of P220 cleavage during picornavirus infection to 2A proteinase sequencing. J. Virol..

[4] van Kuppeveld F.J., Galama J.M., Zoll J., Melchers W.J. (1995). Genetic analysis of a hydrophobic domain of coxsackie B3 virus protein 2B: A moderate degree of hydrophobicity is required for a cis-acting function in viral RNA synthesis. J. Virol..

[5] Saeed M., Kapell S., Hertz N.T., Wu X., Bell K., Ashbrook A.W., Mark M.T., Zebroski H.A., Neal M.L., Flodström-Tullberg M. (2020). Defining the proteolytic landscape during enterovirus infection. PLoS Pathog..

[6] De Jesús-González L.A., Palacios-Rápalo S., Reyes-Ruiz J.M., Osuna-Ramos J.F., Cordero-Rivera C.D., Farfan-Morales C.N., Gutiérrez-Escolano A.L., Del Ángel R.M. (2021). The nuclear pore complex is a key target of viral proteases to promote viral replication. Viruses.

[7] Walker E.J., Younessi P., Fulcher A.J., McCuaig R., Thomas B.J., Bardin P.G., Jans D.A., Ghildyal R. (2013). Rhinovirus 3C protease facilitates specific nucleoporin cleavage and mislocalisation of nuclear proteins in infected host cells. PLoS ONE.

[8] Gustin K.E., Sarnow P. (2001). Effects of poliovirus infection on nucleo-cytoplasmic trafficking and nuclear pore complex composition. EMBO J..

[9] Watters K., Inankur B., Gardiner J.C., Warrick J., Sherer N.M., Yin J., Palmenberg A.C. (2017). Differential disruption of nucleocytoplasmic trafficking pathways by rhinovirus 2A proteases. J. Virol..

[10] Watters K., Palmenberg A.C. (2011). Differential processing of nuclear pore complex proteins by rhinovirus 2A proteases from different species and serotypes. J. Virol..

[11] Jin Y., Zhang R., Wu W., Duan G. (2018). Innate immunity evasion by enteroviruses linked to epidemic hand-foot-mouth disease. Front. Microbiol..

[12] García M.A., Meurs E.F., Esteban M. (2007). The DsRNA protein kinase PKR: Virus and cell control. Biochimie.

[13] Montero H., Trujillo-Alonso V. (2011). Stress granules in the viral replication cycle. Viruses.

[14] Reineke L.C., Dougherty J.D., Pierre P., Lloyd R.E. (2012). Large G3BP-induced granules trigger EIF2α phosphorylation. Mol. Biol. Cell.

[15] Dabo S., Meurs E.F. (2012). DsRNA-dependent protein kinase PKR and its role in stress, signaling and HCV infection. Viruses.

[16] Onomoto K., Jogi M., Yoo J.S., Narita R., Morimoto S., Takemura A., Sambhara S., Kawaguchi A., Osari S., Nagata K. (2012). Critical role of an antiviral stress granule containing RIG-I and PKR in viral detection and innate immunity. PLoS ONE.

[17] Yang X., Hu Z., Zhang Q., Fan S., Zhong Y., Guo D., Qin Y., Chen M. (2019). SG formation relies on EIF4GI-G3BP interaction which is targeted by picornavirus stress antagonists. Cell Discov..

[18] Visser L.J., Langereis M.A., Rabouw H.H., Wahedi M., Muntjewerff E.M., de Groot R.J., van Kuppeveld F.J.M. (2019). Essential role of enterovirus 2A Protease in counteracting counteracting stress granule formation and the induction of type I interferon. J. Virol..

[19] Feng Q., Langereis M.A., Lork M., Nguyen M., Hato S.V., Lanke K., Emdad L., Bhoopathi P., Fisher P.B., Lloyd R.E. (2014). Enterovirus 2Apro targets MDA5 and MAVS in infected cells. J. Virol..

[20] Rasti M., Khanbabaei H., Teimoori A. (2019). An update on enterovirus 71 infection and interferon type I response. Rev. Med. Virol..

[21] Chen B., Wang Y., Pei X., Wang S., Zhang H., Peng Y. (2020). Cellular caspase-3 contributes to EV-A71 2Apro-mediated down- regulation of IFNAR1 at the translation level. Virol. Sin..

[22] Wang L., Chen S., Chang S., Lee Y., Yu C., Chen C., Tseng P., Hsieh C., Chen S., Lin C. (2015). Enterovirus 71 proteins 2A and 3D antagonize the antiviral activity of gamma interferon via signaling attenuation. J. Virol..

[23] Kang J., Pang Z., Zhou Z., Li X., Liu S., Cheng J., Liu P., Tan W., Wang Z., Wang T. (2021). Enterovirus D68 protease 2Apro targets TRAF3 to subvert host innate immune responses. J. Virol..

[24] Kwiatkowski S., Seliga A.K., Vertommen D., Terreri M., Ishikawa T., Grabowska I., Tiebe M., Teleman A.A., Jagielski A.K., Veiga-da-cunha M. (2018). SETD3 protein is the actin-specific histidine N-methyltransferase. eLife.

[25] Wilkinson A.W., Diep J., Dai S., Liu S., Ooi Y.S., Song D., Li T., Horton J.R., Zhang X., Liu C. (2019). SETD3 is an actin histidine methyltransferase that prevents primary dystocia. Nature.

[26] Diep J., Ooi Y.S., Wilkinson A.W., Peters C.E., Foy E., Johnson J.R., Zengel J., Ding S., Weng K.F., Laufman O. (2019). Enterovirus pathogenesis requires the host methyltransferase SETD3. Nat. Microbiol..

[27] Borghese F., Michiels T. (2011). The leader protein of cardioviruses inhibits stress granule assembly. J. Virol..

[28] Porter F.W., Palmenberg A.C. (2009). Leader-induced phosphorylation of nucleoporins correlates with nuclear trafficking inhibition by cardioviruses. J. Virol..

[29] Rabouw H.H., Langereis M.A., Knaap R.C.M., Dalebout T.J., Canton J., Sola I., Enjuanes L., Bredenbeek P.J., Kikkert M., de Groot R.J. (2016). Middle east respiratory coronavirus accessory protein 4a inhibits PKR-Mediated antiviral stress responses. PLoS Pathog..

[30] Visser L.J., Media G.N., Rabouw H.H., de Groot R.J., Langereis M.A., de los Santos T., van Kuppeveld F.J.M. (2019). Foot-and-mouth disease virus leader protease cleaves G3BP1 and G3BP2 and inhibits stress granule formation. J. Virol..

[31] Duke G.M., Palmenberg A.C. (1989). Cloning and synthesis of infectious cardiovirus RNAs containing short, discrete poly(C) tracts. J. Virol..

[32] Kräusslich H.G., Nicklin M.J., Toyoda H., Etchison D., Wimmer E. (1987). Poliovirus proteinase 2A induces cleavage of eucaryotic initiation factor 4F polypeptide P220. J. Virol..

[33] Matunis M.J., Michael W.M., Dreyfuss G. (1992). Characterization and primary structure of the poly(C)-binding heterogeneous nuclear ribonucleoprotein complex K protein. Mol. Cell. Biol..

[34] Michael W.M., Eder P.S., Dreyfuss G. (1997). The K nuclear shuttling domain: A novel signal for nuclear import and nuclear export in the HnRNP K protein. EMBO J..

[35] Wu S., Wang Y., Lin L., Si X., Wang T., Zhong X., Tong L., Luan Y., Chen Y., Li X. (2014). Protease 2A induces stress granule formation during coxsackievirus B3 and enterovirus 71 infections. Virol. J..

[36] Honda K., Yanai H., Takaoka A., Taniguchi T. (2005). Regulation of the type I IFN induction: A current view. Int. Immunol..

[37] Fitzgerald K.D., Chase A.J., Cathcart A.L., Tran G.P., Semler B.L. (2013). Viral proteinase requirements for the nucleocytoplasmic relocalization of cellular splicing factor SRp20 during picornavirus infections. J. Virol..

[38] Molla A., Paul A.V., Schmid M., Jang S.K., Wimmer E. (1993). Studies on dicistronic polioviruses implicate viral proteinase 2Apro in RNA replication. Virology.

[39] Yang X., Hu Z., Fan S., Zhang Q., Zhong Y., Guo D., Qin Y., Chen M. (2018). Picornavirus 2A protease regulates stress granule formation to facilitate viral translation. PLoS Pathog..

[40] Delhaye S., van Pesch V., Michiels T. (2004). The leader protein of theiler’s virus interferes with nucleocytoplasmic trafficking of cellular proteins. J. Virol..

[41] Bardina M.V., Lidsky P.V., Sheval E.V., Fominykh K.V., van Kuppeveld F.J.M., Polyakov V.Y., Agol V.I. (2009). Mengovirus-induced rearrangement of the nuclear pore complex: Hijacking cellular phosphorylation machinery. J. Virol..

[42] Porter F.W., Bochkov Y.A., Albee A.J., Wiese C., Palmenberg A.C. (2006). A picornavirus protein interacts with Ran-GTPase and disrupts nucleocytoplasmic transport. Proc. Natl. Acad. Sci. USA.

[43] Ciomperlik J.J., Basta H.A., Palmenberg A.C. (2015). Three cardiovirus leader proteins equivalently inhibit four different nucleocytoplasmic trafficking pathways. Virology.

[44] Porter F.W., Brown B., Palmenberg A.C. (2010). Nucleoporin phosphorylation triggered by the encephalomyocarditis virus leader protein is mediated by mitogen-activated protein kinases. J. Virol..

[45] Sorgeloos F., Peeters M., Hayashi Y., Borghese F., Capelli N., Drappier M., Cesaro T., Colau D., Stroobant V., Vertommen D. (2022). A case of convergent evolution: Several viral and bacterial pathogens hijack RSK kinases through a common linear motif. Proc. Natl. Acad. Sci. USA.

