# Supplementary material for: Proteolytic Activities of Enterovirus 2A Do Not Depend on Its Interaction with SETD3
Source: Viruses. 2022 Jun 22;14(7):1360. doi: 10.3390/v14071360 (PMC9318592; doi:10.3390/v14071360)

Figure S1. CVB3 infection in HeLa wt and SETD3^{KO} cells. Cells were infected with CVB3-Rluc viruses at MOI of 0.1. Lysis buffer was added and *Renilla* luciferase activity was tested to evaluate the virus replication at 2, 4, 6 and 8 hpi.

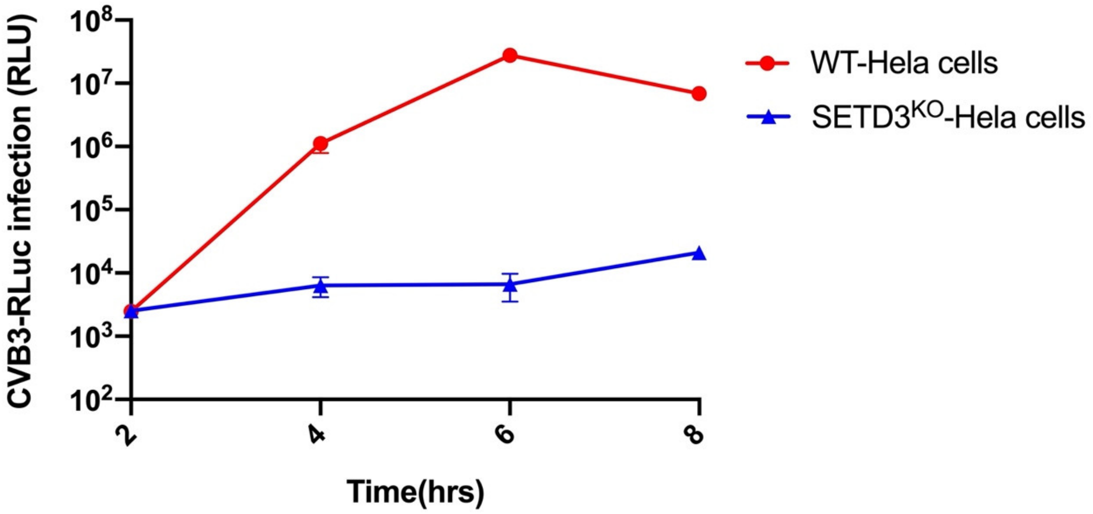

Supplement: Supplementary file 1 [file viruses-14-01360-s001.zip › viruses-1754063-supplementary.pdf]
